# Impaired formation of high-order gephyrin oligomers underlies gephyrin dysfunction-associated pathologies

**DOI:** 10.1016/j.isci.2021.102037

**Published:** 2021-01-07

**Authors:** Seungjoon Kim, Mooseok Kang, Dongseok Park, Ae-Ree Lee, Heinrich Betz, Jaewon Ko, Iksoo Chang, Ji Won Um

**Affiliations:** 1 Department of Brain and Cognitive Sciences, Daegu Gyeongbuk Institute of Science and Technology (DGIST), Daegu 42988, Korea; 2 Core Protein Resources Center, DGIST, Daegu 42988, Korea; 3 Max Planck Institute for Medical Research, Jahnstrasse 29, 69120 Heidelberg, Germany; 4 Supercomputing Bigdata Center, DGIST, Daegu 42988, Korea

**Keywords:** Molecular Biology, Neuroscience, Structural Biology

## Abstract

Gephyrin is critical for the structure, function, and plasticity of inhibitory synapses. Gephyrin mutations have been linked to various neurological disorders; however, systematic analyses of the functional consequences of these mutations are lacking. Here, we performed molecular dynamics simulations of gephyrin to predict how six reported point mutations might change the structural stability and/or function of gephyrin. Additional *in silico* analyses revealed that the A91T and G375D mutations reduce the binding free energy of gephyrin oligomer formation. Gephyrin A91T and G375D displayed altered clustering patterns in COS-7 cells and nullified the inhibitory synapse-promoting effect of gephyrin in cultured neurons. However, only the G375D mutation reduced gephyrin interaction with GABA_A_ receptors and neuroligin-2 in mouse brain; it also failed to normalize deficits in GABAergic synapse maintenance and neuronal hyperactivity observed in hippocampal dentate gyrus-specific gephyrin-deficient mice. Our results provide insights into biochemical, cell-biological, and network-activity effects of the pathogenic G375D mutation.

## Highlights

• Gephyrin G375D reduces the formation of E- domain-mediated gephyrin oligomers
• Gephyrin G375D impairs GABAergic synapse maintenance *in vivo*
• Gephyrin G375D increases susceptibility to kainic acid-induced seizures *in vivo*
• Gephyrin G375D displays reduced interactions with GABA_A_ receptors and Nlgn2 *in vivo*

## Graphical Abstract

**Figure:**
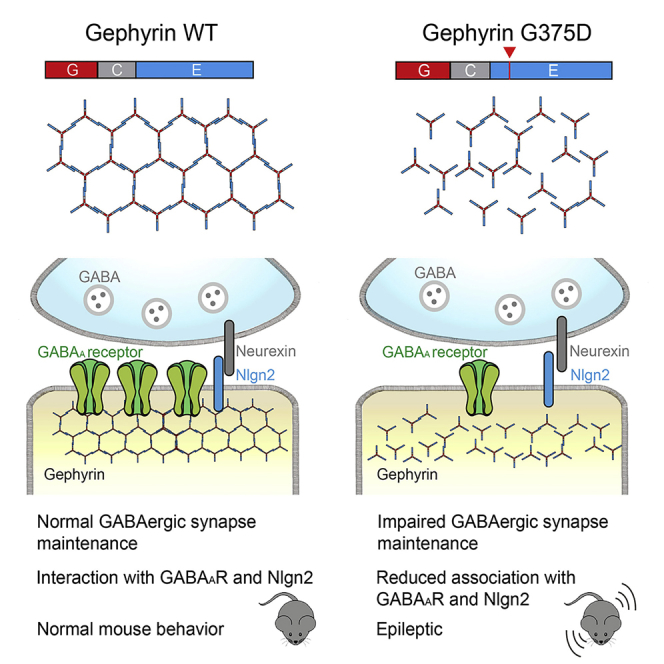

## Introduction

Recent research in sequencing and genomics has shown clear associations of numerous synaptic genes with a variety of neuropsychiatric and neurodevelopmental diseases, including autism spectrum disorders (ASDs), schizophrenia and epilepsy, giving rise to the ‘synaptopathy’ concept (Medland et al., 2014; Sestan and State, 2018; State and Levitt, 2011). However, in addition to the complex inheritance pattern of the culprit synaptic genes implicated in these disorders, complexities in genetic causation profiles have challenged our ability to establish how dysfunction(s) of risk gene-encoded proteins at synapses precisely contribute to disease onset and/or progression (Heinzen et al., 2015). In cases of highly variable point mutants, empirical approaches combined with *in silico* analyses can be used *a priori* to investigate whether these mutations are loss- or gain-of-function mutations and to determine how altered properties might affect specific aspects of synapse development and animal behavior [e.g. (Anastasia et al., 2013; Bath and Lee, 2006; Falivelli et al., 2012; Kang et al., 2016; Riccardi et al., 2019; Südhof, 2017)].

Gephyrin contributes to the accumulation of GABA_A_ receptors at postsynaptic sites by interacting with several key GABAergic synapse-specific proteins, enabling efficient inhibitory synaptic transmission (Betz, 1998; Choii and Ko, 2015; Fritschy et al., 2008; Groeneweg et al., 2018; Pizzarelli et al., 2019; Tyagarajan and Fritschy, 2014). In addition, gephyrin is known to be required for synaptic clustering of glycine receptors (GlyRs) in the spinal cord and for molybdoenzyme activity in non-neuronal tissues (Feng et al., 1998; Kirsch et al., 1993; Stallmeyer et al., 1999). Gephyrin is composed of three functional domains: an N-terminal G-domain, a central C-domain, and a C-terminal E-domain (Choii and Ko, 2015; Kim et al., 2006; Pizzarelli et al., 2019; Sola et al., 2004). At postsynaptic sites, gephyrin assembles through G- and E-domain–mediated interactions into a complex submembranous lattice that is dynamically regulated by a number of posttranslational modifications and interactions with other binding proteins (Choii and Ko, 2015; Groeneweg et al., 2018; Tyagarajan and Fritschy, 2014). The lattice formed by gephyrin is multimeric (Saiyed et al., 2007), with the G-domain-mediated trimeric form being prominent in bacteria (Sander et al., 2013). Trimerization of G-domains and dimerization of E-domains are considered essential for the ability of gephyrin to form hexagonal membrane-associated scaffolds and recruit GlyRs and GABA_A_ receptors (GABA_A_Rs) to developing synapses (Calamai et al., 2009; Kneussel and Betz, 2000; Saiyed et al., 2007; Sola et al., 2004). Not surprisingly, gephyrin has been implicated in various brain disorders, including autism, schizophrenia, Alzheimer's disease, and epilepsy (Agarwal et al., 2008; Dejanovic et al., 2014; Forstera et al., 2010; Kiss et al., 2016; Lionel et al., 2013). Sequencing studies have suggested that irregular alternative splicing within the G-domain coding region, large exonic deletions, and missense mutations are linked to neuronal or metabolic dysfunction of gephyrin (Dejanovic et al., 2014, 2015; Forstera et al., 2010; Lionel et al., 2013). However, whether the resulting changes in gephyrin structure directly affect the formation of intact hexagonal multimeric lattices or are linked to GABAergic synapse development has not been investigated. A heterozygous missense mutation, p.Gly375Asp (G375D) in the gephyrin gene, *GPHN*, has been reported to disrupt the function of endogenous gephyrin proteins in neurons, resulting in reduced binding affinity for GABA_A_Rs and GlyRs as well as impaired molybdenum cofactor synthesis activity (Dejanovic et al., 2015). However, apart from these phenomenological descriptions of gephyrin mutations based on a set of functional assays, little is understood about whether and how the reported mutations actually alter gephyrin's oligomerization properties and interactions with other synaptic proteins to contribute to changes in gephyrin scaffold formation.

In the present study, we performed atomic-scale molecular dynamics (MD) simulations combined with thermodynamic integration (TI) calculations for wild-type (WT) gephyrin and a subset of gephyrin missense mutants associated with ASDs or epileptic encephalopathy, to test the hypothesis that these mutations affect the structural stability of the multimeric gephyrin complex. The gephyrin mutants, A91T and G375D, which displayed altered binding free energies *in silico*, were chosen for further functional analyses. Remarkably, only G375D, and not A91T, exerted loss-of-function phenotypes *in vivo*, including an abnormally heightened epileptogenic potential. Our study provides insights into gephyrin dysfunction-associated synaptopathies and underscores the potential of supercomputing-based simulations to predict deleterious effects of synaptic protein mutations.

## Results

### Molecular dynamics simulation-based prediction of conformational changes produced by gephyrin missense mutations associated with neurological disorders

Six missense mutations of gephyrin—V43L, A91T, G375D, G578A, G578S, and D697N—have previously been linked to ASDs and/or epileptic encephalopathy (Dejanovic et al., 2015; Lionel et al., 2013). Among these, gephyrin G375D has been shown to disrupt neuronal and metabolic functions of gephyrin (Dejanovic et al., 2015). Specifically, G375D acts in a dominant-negative manner to inhibit complex formation between gephyrin and GABA_A_Rs or GlyRs, and further compromises molybdenum cofactor synthesis activity (Dejanovic et al., 2015). Although gephyrin G375D was demonstrated to form oligomers with gephyrin WT by size-exclusion chromatography, it has not been clarified whether this mutation impairs formation of gephyrin multimers by severing the structure of the E-domain, where the G375 residue is located.

**Figure 1:**
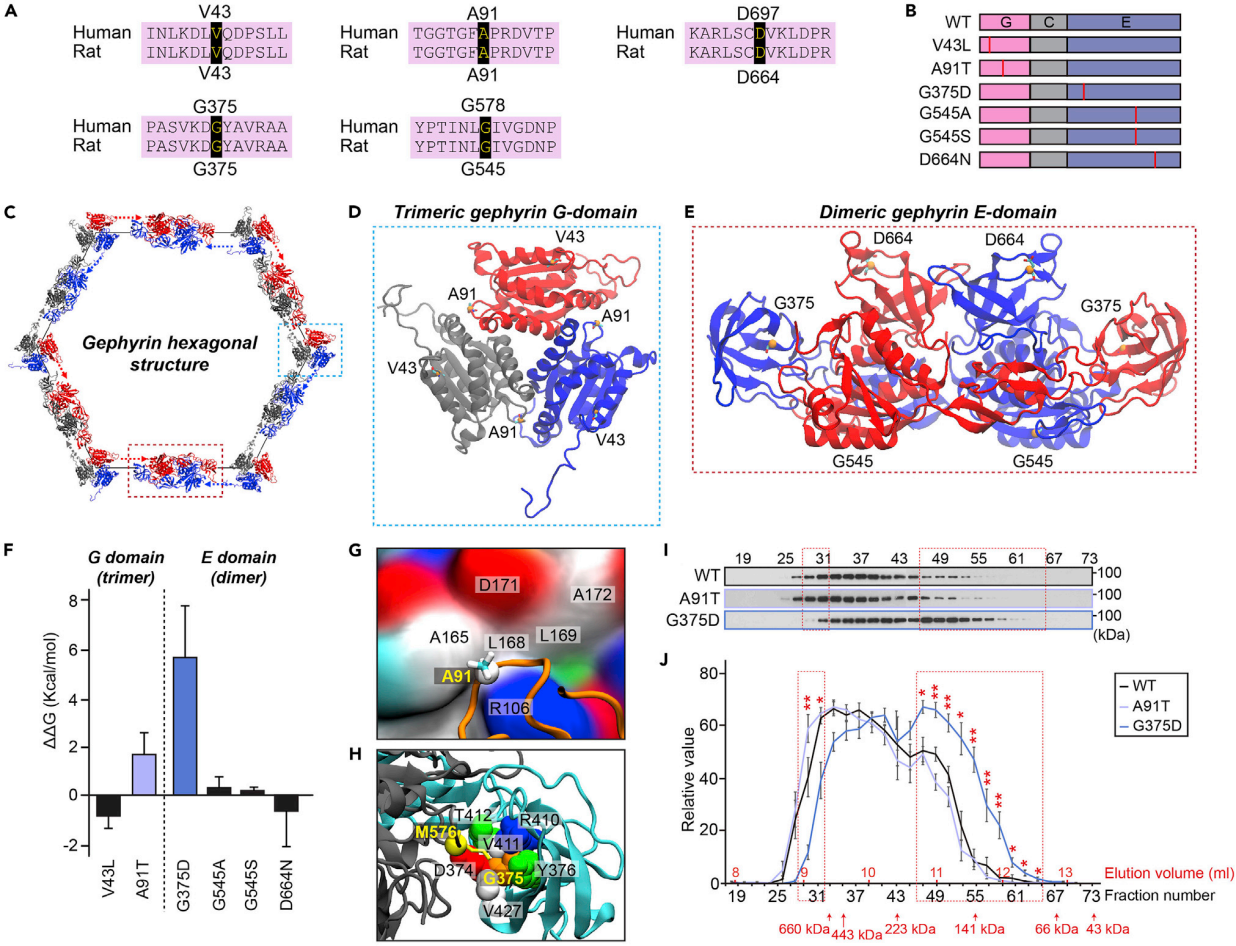
A human gephyrin missense mutation associated with epileptic encephalitis alters multimer binding stability (A) Alignment and conservation between human gephyrin- and rat gephyrin residues that are mutated in human patients with epileptic encephalopathy or ASDs. (B) Schematic diagrams of gephyrin WT and its mutants. Abbreviations: G, G-domain; C, C-domain; E, E-domain. (C) Schematic depiction of gephyrin hexagonal lattices. (D) Structure of G-domain trimers and position of point mutations in gephyrin. (E) Structure of E-domain dimers and position of point mutations in gephyrin. (F) Multimer binding free energy difference between gephyrin WT and the indicated point mutants, as calculated by thermodynamic integration analyses. Numerical values per mutation points were averaged. Data are represented as means ± SDs. (G) A91 and hydrophobic pockets in the binding interface between gephyrin G-domains. (H) G375 positioned inside of the β-strand bundle at the binding interface between chains. Bead and surface colors indicate the following: white, hydrophobic; green, hydrophilic; red, negatively charged; blue, positively charged; and yellow, methionine. (I) Extracts of HEK293T cells transfected with untagged gephyrin WT or its mutants (A91T or G375D) were fractionated on a gel filtration column. Fractions were analyzed by immunoblotting using anti-gephyrin antibodies. (J) Quantification of expression levels of gephyrin WT and its mutants (A91T or G375D) in each fraction. Data are means ± SEMs (∗p < 0.05, ∗∗p < 0.01; WT versus G375D; Mann-Whitney *U* test; n = 5/group).

To address whether pathogenic gephyrin mutations located in G- or E-domains affect the stability of the hexagonal gephyrin scaffold, we explored potential accompanying conformational changes by performing supercomputing MD simulations of gephyrin G-domain trimers and E-domain dimers. V43, A91, G375, G578, and D697 residues in human gephyrin are conserved in rat gephyrin and correspond to V43, A91, G375, G545, and D664 residues, respectively, of rat gephyrin (Figure 1A). For structural modeling studies and functional assays, we produced the rat gephyrin G-domain (amino acids 1–188) mutants V43L and A91T and E-domain (amino acids 318–736) G375D, G545A, G545S, and D664N by replacing these conserved residues with the corresponding amino acids found in human patients (Figure 1B). The G-domain trimer has a 3-fold symmetry in which the α-helix and loops are involved in the interface between monomers (Schwarz et al., 2001; Sola et al., 2001) (Figures 1C and 1D). V43 is located on the outer α-helix of the trimer and A91 is located near the binding interface with other chains (Figure 1D). Our MD simulations of V43L and A91T mutants indicated no conformational change in the monomer or trimer. It also revealed no difference in structural fluctuations of residues during a 0.5–1 μs simulation, except for the terminal loop, and showed no structural difference between WT and mutant proteins, even near the mutated residues (Figure S1A).

The E-domain dimer has 2-fold symmetry, and each monomer consists of four subdomains, of which the receptor-binding subdomains (residues 654–736) are known to bind to GABA_A_Rs and GlyRs (Kim et al., 2006; Maric et al., 2014; Sola et al., 2004). G375 is located in the β-strand bundle subdomain (residues 367–463) on one side, D664 is located in the receptor-binding subdomain, and G545 is located on the other side (Figures 1C and 1E). MD simulations performed on gephyrin WT, G375D, G545A, G545S, and D664N mutants showed large structural fluctuations in the β-strand bundle subdomain of both WT and mutants (Figure S1B). However, this type of fluctuation was observed in all MD trajectories, regardless of mutation status, and there were no conformational changes in monomers or dimers at or near the mutation interface. We initially expected that a single mutation could alter the structure of gephyrin or influence the trimeric and/or dimeric structure but our MD simulation results predicted no significant structural changes.

### Calculation of differences in binding stability between gephyrin WT and mutants using TI MD simulations

Because single point mutations in gephyrin conferred no significant conformational changes on monomeric or multimeric structures of gephyrin (Figure S1), we next examined effects of the mutation on the binding stability of the multimer. Using TIMD simulations to calculate the binding free energy difference between WT and mutants of the G-domain trimer and E-domain dimer, we found that the binding free energies of the A91T and G375D mutants, in which the mutations occur near the binding interface, were significantly changed compared with that of the WT (Figure 1F). Specifically, the binding free energy difference of A91T relative to the WT was determined to be 5.1 ± 2.7 kcal/mol, or 1.7 kcal/mol per mutation point (Figure 1F). A91 is a hydrophobic residue located at the binding interface of the G-domain trimer, where it is exposed to the outside and bound to a hydrophobic pocket consisting of A165, L168, L169, and A172 (Figure 1G). Mutation of this alanine to the hydrophilic threonine weakens hydrophobic interaction with this pocket, reducing the binding stability of the A91T G-domain trimer compared with that of the WT. However, given thermal fluctuation effects, the binding free energy difference of A91T might be non-significant.

For G375D, the binding free energy difference relative to WT was determined to be 11.3 ± 4.3 kcal/mol, a highly significant difference of 5.7 kcal/mol per mutation point (Figure 1F). G375 is located at the center of the β-strand bundle subdomain and lies close to the binding interface of the GABA_A_R- and GlyR-binding subdomain of another E-domain (Figure 1H). At the binding interface of the two subdomains, the hydrophobic side chain of M576 in the receptor-binding subdomain is fit into the pocket of the β-strand bundle subdomain consisting of D374, G375, Y376, R410, V411, T412, and V427. Changing this central glycine to a negatively charged aspartic acid breaks the electrostatic equilibrium and spatial conditions of the binding pocket, decreasing the binding stability of the E-domain dimer and weakening contacts of the monomers. Taken together, our MD simulations revealed that A91T and G375D mutations lower the binding affinity of the multimer rather than directly altering the entire structure, thereby affecting the stability of gephyrin hexagonal structures.

To validate these structural predictions experimentally, we performed gel filtration chromatography to test whether A91T and G375D mutations affect the abundance of gephyrin oligomers formed upon expression in HEK293T cells (Figures 1I and 1J). Immunoblot analyses of each fraction revealed the similar elution patterns for gephyrin A91T and gephyrin WT. In contrast, under the same conditions a significant fraction of gephyrin G375D was shifted to lower molecular weight positions (Figures 1I and 1J). Specifically, gephyrin G375D abundance was significantly lower than that of WT in > 600 kDa fractions (fractions 27–33) but was more abundant in < 200-kDa fractions (fractions 49–61) (Figures 1I and 1J). These results suggest that gephyrin G375D higher-order oligomers are less frequent or are less stable, than those of the WT protein, in keeping with the results of our TIMD simulations.

### Gephyrin G375D is defective in promoting GABAergic synapse formation *in vitro*

**Figure 2:**
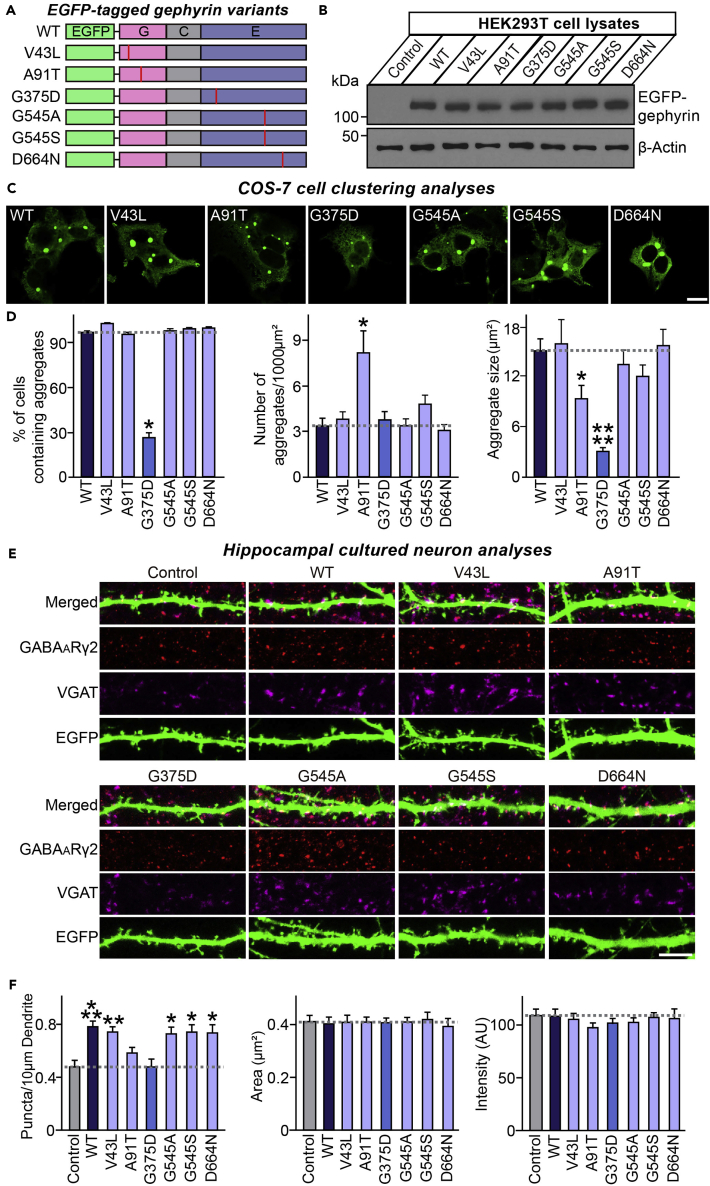
Gephyrin G375D, but not A91T, is defective in promoting GABAergic synapse formation in cultured hippocampal neurons (A) Schematic diagrams of gephyrin WT and its mutants. Abbreviations: G, G-domain; C, C-domain; E, E-domain. (B) Immunoblotting of lysates from HEK293T cells transfected with EGFP-tagged gephyrin WT or its mutants using anti-gephyrin antibodies. An anti-β-actin antibody was used as a normalization control. (C) Representative images of COS-7 cells transfected with EGFP-gephyrin WT or its mutants. Scale bar, 10 μm (applies to all images). (D) Quantification of the percentage of COS-7 cells with large cytoplasmic intracellular aggregates, the number of aggregates per 1000 μm^2^, and average aggregate size. Data are presented as means ± SEMs from three independent experiments (∗p < 0.05, ∗∗∗∗p < 0.0001; non-parametric ANOVA with Kruskal-Wallis test followed by *post hoc* Dunn's multiple comparison test). (E) Representative images of cultured hippocampal neurons transfected at DIV10 with EGFP alone (Control) or cotransfected with EGFP alone and EGFP-tagged gephyrin WT or its mutants, and analyzed at DIV14 by triple-immunofluorescence staining using anti-GABA_A_Rγ2 (red), anti-VGAT (magenta) and anti-EGFP (green) antibodies. Scale bar, 10 μm (applies to all images). (F) Summary graphs of the effects of overexpressing gephyrin WT or its mutants on GABA_A_Rγ2^+^VGAT^+^ puncta density (left), GABA_A_Rγ2^+^VGAT^+^ puncta size (middle), and GABA_A_Rγ2^+^VGAT^+^ puncta intensity (right). Data are presented as means ± SEMs from three independent experiments (2–3 dendrites per transfected neurons were analyzed and group-averaged; ∗p < 0.05, ∗∗p < 0.01, ∗∗∗p < 0.001; non-parametric ANOVA with Kruskal-Wallis test followed by *post hoc* Dunn's multiple comparison test).

To assess whether the mutations in the *GPHN* gene described above alter the clustering properties of gephyrin, we generated enhanced green fluorescence protein (EGFP)-tagged constructs of gephyrin WT and its mutants (Figure 2A). Immunoblot analyses showed that expression levels of the tagged gephyrin point mutants were comparable to those of WT (Figure 2B). Recombinant gephyrin is known to form large intracellular aggregates in non-neuronal cells (Kins et al., 2000). As previously reported, EGFP-gephyrin WT consistently formed large intracellular clusters when expressed in COS-7 cells (Figure 2C). Under the same conditions, exogenously expressed gephyrin A91T formed significantly more, but smaller, aggregates, whereas G375D produced tiny aggregates in only 30% of transfected cells (Figures 2C and 2D). The other gephyrin mutants examined, V43L, G545A, G545S, and D664N, generated aggregates similar to those formed by gephyrin WT (Figures 2C and 2D). These results are consistent with the reduced oligomerization potential of A91T and G375D, but unchanged oligomerization properties of the other mutants.

To determine whether the gephyrin mutations indicated above affect GABAergic synaptic targeting of gephyrin mutants, we transfected cultured hippocampal neurons with EGFP-tagged gephyrin WT or the indicated mutants at 10 days *in vitro* (DIV10), and immunostained transfected neurons for the GABAergic presynaptic marker, VGAT (vesicular GABA transporter), and the postsynaptic GABA_A_ receptor γ2 subunit (GABA_A_Rγ2) at DIV14. An assessment of the subcellular localization of gephyrin mutants, visualized by monitoring GFP immunofluorescence, revealed that gephyrin WT was mainly distributed to GABAergic synaptic sites, whereas both A91T and G375D were diffusely distributed along dendrites of the transfected neurons, compared to gephyrin WT and other mutants (Figure S2). Next, to determine whether gephyrin mutations also compromise the ability of gephyrin to promote GABAergic synapse formation, we transfected cultured hippocampal neurons with EGFP-tagged gephyrin WT or mutants at DIV10, together with EGFP empty vector to clearly visualize the dendritic segments and immunostained transfected neurons for the GABAergic synaptic markers, VGAT and GABA_A_Rγ2, at DIV14. Overexpression of gephyrin WT in cultured hippocampal neurons significantly increased the density, but not the size or fluorescence intensity of VGAT^+^/GABA_A_Rγ2^+^ puncta, as compared with neurons expressing EGFP alone (Figures 2E and 2F). Notably, overexpression of the mutants, V43L, G545A, G545S, or D664N, increased VGAT^+^/GABA_A_Rγ2^+^ puncta density to a similar extent as gephyrin WT, whereas overexpression of G375D or A91T had no such effect on synaptic marker puncta (Figures 2E and 2F). Collectively, these results are consistent with the idea that the gephyrin G375D mutation impairs the formation of or destabilizes higher-order gephyrin oligomers, thereby disrupting clustering of postsynaptic GABA_A_ receptors.

### Gephyrin G375D reduces GABAergic synapse numbers in hippocampal dentate gyrus granule neurons of adult mice

**Figure 3:**
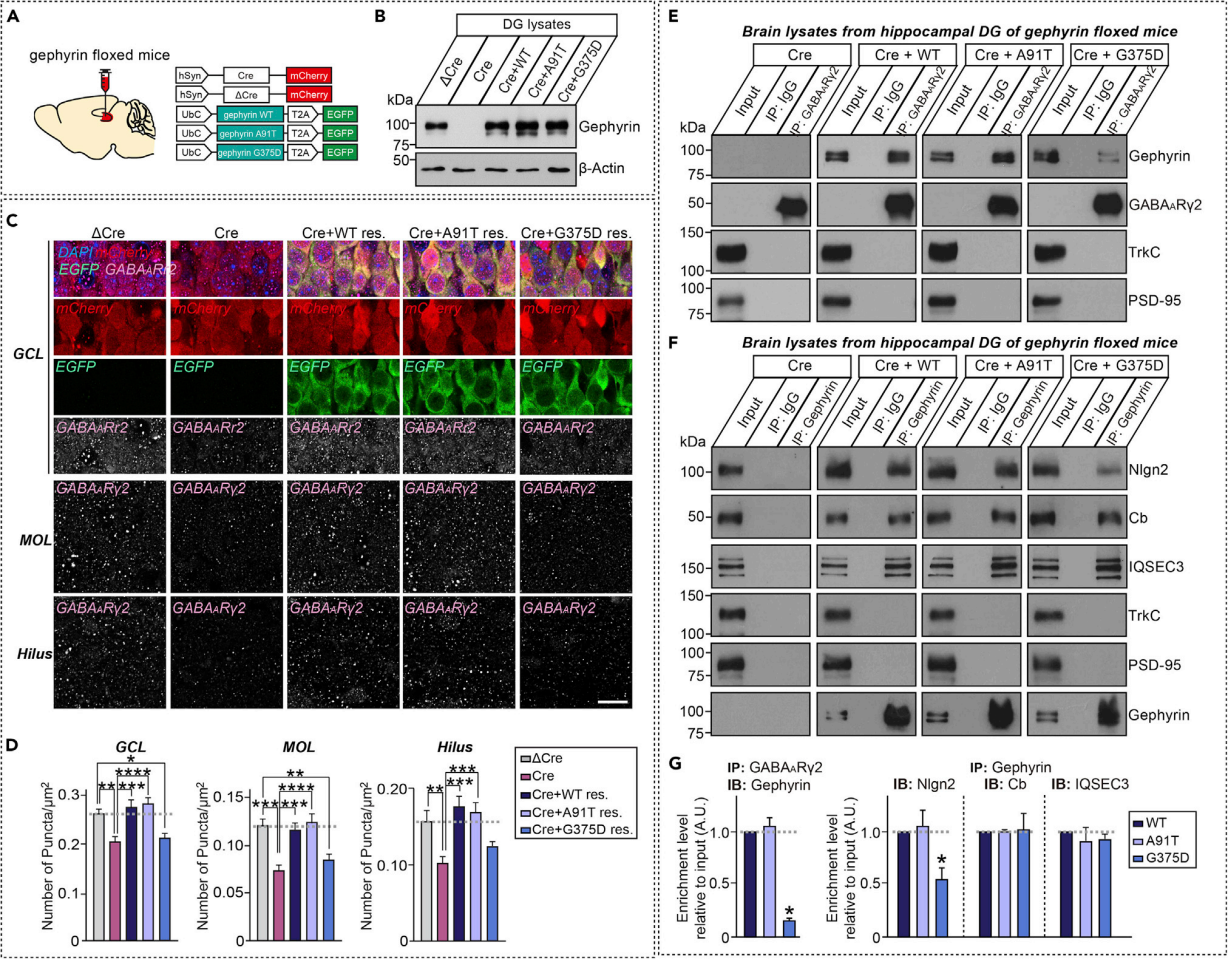
Gephyrin G375D impairs promotion of GABAergic synapse maintenance *in vivo* (A) Schematic diagram of AAV vectors expressing Cre or ΔCre and WT gephyrin and its mutants (A91T and G375D) used for stereotactic injection into the DG of gephyrin floxed mice. (B) Immunoblotting analyses with gephyrin antibodies validating gephyrin knockout and expression of gephyrin rescue constructs *in vivo.* Lysates from mouse brains stereotactically injected with AAVs were collected and immunoblotted with anti-gephyrin antibodies. An anti-β-actin antibody was used as a normalization control. (C) Representative images showing GABA_A_Rγ2^+^ puncta in the DG of mice stereotactically injected with the indicated AAVs. Scale bar, 20 μm (applies to all images). Abbreviations: MOL, molecular layer; GCL, granule cell layer. (D) Quantification of the density of GABA_A_Rγ2^+^ puncta per tissue area. Data are presented as means ± SEMs (n = 4 mice each after averaging data from 5 sections/mouse; ∗p < 0.05, ∗∗p < 0.01, ∗∗∗p < 0.001, ∗∗∗∗p < 0.0001; non-parametric ANOVA with Kruskal-Wallis test followed by *post hoc* Dunn's multiple comparison test). (E) Synaptosomal fractions of adult brains from gephyrin floxed mice stereotactically injected with the indicated AAV viruses were immunoprecipitated with anti-GABA_A_Rγ2 antibodies and immunoblotted with anti-GABA_A_Rγ2, anti-gephyrin, anti-TrkC or anti-PSD-95 antibodies. An equal amount of rabbit IgG was used as a negative control. Input, 5%. (F) Synaptosomal fractions of adult brains from gephyrin floxed mice stereotactically injected with the indicated AAV viruses were immunoprecipitated with an anti-gephyrin antibody and immunoblotted with the indicated antibodies. An equal amount of mouse IgG was used as a negative control. Input, 5%. (G) Quantification of coimmunoprecipitated synaptic proteins in (E) or (F), normalized to controls. Data are means ± SEM from three independent experiments. (∗p < 0.05; non-parametric Kruskal-Wallis test with Dunn's *post hoc* test).

To extend the observations made with the gephyrin mutants A91T and G375D to neurons *in vivo*, we used gephyrin floxed mice (O'Sullivan et al., 2016) stereotactically injected in the hippocampal dentate gyrus (DG) with adeno-associated viruses (AAVs) expressing either mCherry-fused nuclear Cre-recombinase or a non-functional mutant version of Cre-recombinase (ΔCre), as a control, to generate DG-specific gephyrin-knockout (KO) mice (Figure 3A). To determine whether the effects of gephyrin deletion in the DG on GABAergic synapse maintenance could be rescued, we also co-injected AAVs expressing Cre-recombinase with AAVs expressing EGFP-fused WT or mutant (A91T or G375D) gephyrin (Figure 3A). Targeted delivery of AAVs into the DG of mice was verified (Figures S3A and S3B), and AAV coinfection efficiency was comparable among all groups (Cre and gephyrin WT, 98.05 ± 0.70%; Cre and gephyrin A91T, 97.58 ± 0.65%; and Cre and gephyrin G375D, 97.95 ± 0.79%) (Figure S3C). Expression of Cre-recombinase and gephyrin WT or mutant proteins was validated by immunoblotting AAV-infected DG brain tissues with anti-gephyrin antibodies (Figure 3B).

We performed immunohistochemical analyses to probe whether the loss of gephyrin affects GABAergic synapse numbers in the DG (Figures 3C, 3D, S4, and S5). Quantitative immunofluorescence analyses revealed significant decreases in the puncta densities of GABA_A_Rγ2 and VGAT in the DG granular cell layer, molecular layer and hilus upon DG-specific Cre expression (Figures 3C, 3D, and S5). This is consistent with the interpretation that gephyrin contributes to the maintenance of GABAergic synapses in adult hippocampal DG neurons. The decreases in GABA_A_Rγ2 and VGAT densities observed in DG-specific gephyrin-KO mice were both rescued by expression of gephyrin WT or A91T but not by expression of mutant G375D (Figure 3C, 3D, and S5). These results indicate that the gephyrin mutation G375D, but not A91T, impairs the function of gephyrin in maintaining GABAergic synapse numbers *in vivo*.

To substantiate whether A91T or G375D mutations affect interactions of gephyrin with known binding proteins (Choii and Ko, 2015), we performed coimmunoprecipitation analyses using hippocampal DG lysates from gephyrin-KO mice expressing Cre-recombinase and/or gephyrin WT, A91T, or G375D. These analyses revealed that GABA_A_Rγ2 bound WT gephyrin or A91T to a similar extent but showed much less binding to G375D (Figures 3E and 3G). Similarly, association of the GABAergic synapse-specific adhesion protein, Nlgn2, with gephyrin G375D was significantly reduced compared with that of WT or A91T (Figures 3F and 3G). In contrast, amounts of the established gephyrin interaction partners, collybistin (Cb) and IQSEC3, were not affected by either gephyrin missense mutation, suggesting that the G375D mutation selectively disengages gephyrin from GABA_A_ receptors and Nlgn2 *in vivo*. Control coimmunoprecipitation experiments performed in parallel showed no association of GABA_A_Rγ2 or gephyrin with TrkC or PSD-95- (Figures 3E and 3F).

### Gephyrin G375D fails to normalize enhanced seizure susceptibility induced by a gephyrin deficiency

**Figure 4:**
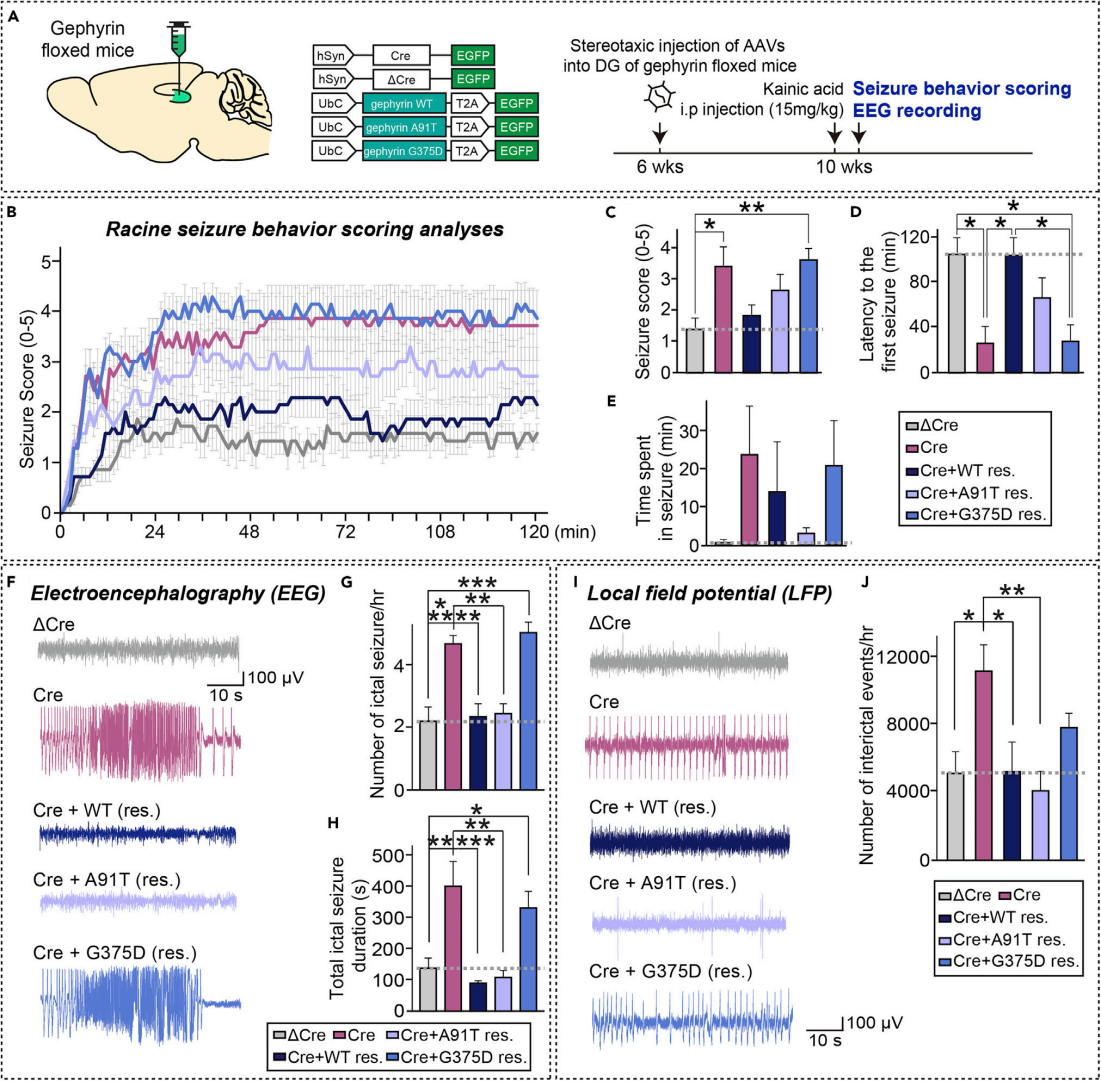
Gephyrin G375D mutant fails to rescue increased seizure susceptibility in DG-specific gephyrin-cKO mice (A) Schematic diagram of AAV vectors expressing Cre or ΔCre and WT gephyrin or its mutants (A91T and G375D) used for stereotactic injection into the DG of gephyrin floxed mice. Experimental scheme for seizure scoring and EEG recordings. The DG region of the hippocampus of ~6-week-old gephyrin floxed mice was bilaterally injected with AAVs-ΔCre or Cre, or co-injected with Cre- and gephyrin-WT–expressing AAVs (Cre + WT res.) or gephyrin-mutant–expressing AAVs (Cre + A91T res. or Cre + G375D res.). Mice were intraperitoneally administered KA 2 weeks after AAV injections, after which seizures were scored and EEGs were recorded. (B) KA-induced seizures in mice injected with the indicated AAVs were scored every 3 min for a total of 120 min, as described in Supplemental Information. Data are presented as means ± SEMs (ΔCre, n = 7 mice; Cre, n = 7 mice; Cre + WT [res.], n = 7 mice; Cre + A91T [res.], n = 7 mice; and Cre + G375D [res.], n = 7 mice; ∗p < 0.05, ∗∗p < 0.01, ∗∗∗p < 0.001 vs. control; Kruskal-Wallis test followed by Dunn's *post hoc* test). (C) Quantification of mean score values under each experimental condition. Data are presented as means ± SEMs (n = 7 mice/condition; ∗p < 0.05, ∗∗p < 0.01 vs. control; Kruskal-Wallis test followed by Dunn's *post hoc* test). (D) Quantification of latency to the first seizure after KA administration under each condition. Data are presented as means ± SEMs (n = 7 mice/condition; ∗p < 0.05; Kruskal-Wallis test followed by Dunn's *post hoc* test). (E) Quantification of time spent in seizure under each condition. Data are presented as means ± SEMs (n = 7 mice/condition). (F) Representative EEG traces of ictal-like seizures recorded from the cortex under the indicated experimental conditions. (G and H) Quantification of the number of ictal-like seizures (G) and total duration of ictal-like seizures (H) per hour under each condition. Data are presented as means ± SEMs (n = 8–11 mice/condition; ∗p < 0.05, ∗∗p < 0.01, ∗∗∗p < 0.001 vs. control; Kruskal-Wallis test followed by Dunn's *post hoc* test). (I) Representative LFP traces of inter-ictal events recorded from the DG under the indicated experimental conditions. (J) Quantification of the number of inter-ictal events under each condition. Data are presented as means ± SEMs (n = 5–6 mice/condition; ∗p < 0.05, ∗∗p < 0.01 vs. control; Kruskal-Wallis test followed by Dunn's *post hoc* test).

We next sought to determine whether gephyrin G375D influences epileptogenicity. To this end, we employed an acute kainic acid (KA)-induced epileptic mouse model in which mice stereotactically injected with the indicated AAVs were intraperitoneally administered KA (15 mg/kg), and then monitored for seizure-related behaviors by video recording (Figure 4A). The severity of KA-induced convulsive seizures was assessed by scoring responses using a revised Racine's scale, which ranks behavior from 0 (no abnormal behavior) to 5 (death) (Racine, 1972) (Figures 4B–4E). In keeping with a previous report that gephyrin deletion in forebrain neurons induces severe seizures (O'Sullivan et al., 2016), average seizure scores for 120 min after KA administration were ~2.45 fold higher in DG-specific gephyrin-KO mice than in control mice (Figures 4B and 4C). The increased seizure susceptibility observed in gephyrin-KO mice was largely normalized by coexpression of gephyrin WT and, to a lesser extent, by coexpression of A91T, but not G375D (Figures 4B–4E). In addition, gephyrin-KO mice exhibited a shorter seizure latency and prolonged seizure duration, effects that were also reduced or abolished by expression of gephyrin WT or A91T, but not G375D (Figures 4Dand 4E).

In line with these differential behavioral responses, electroencephalography (EEG) and local field potential (LFP) recordings revealed higher-amplitude and longer-lasting KA-induced ictal activity in DG-specific gephyrin-KO mice compared with control mice (Figures 4F–4J). The number of ictal seizure events per hour and total ictal seizure duration, determined from EEG activity, were significantly increased in gephyrin-KO mice compared with control mice, effects that were again completely rescued by expression of gephyrin WT or A91T but not G375D (Figures 4F–4H). Moreover, the number of inter-ictal spikes, quantified from 240 min of LFP recordings, was higher in DG-specific gephyrin-KO mice than in control mice (Figures 4I and 4J). This increased frequency of inter-ictal spikes in DG-specific gephyrin-KO mice was similarly normalized by expression of gephyrin WT or A91T, but not G375D (Figures 4I and 4J). Taken together, these results indicate that the gephyrin G375D mutation compromises the excitation/inhibition balance of hippocampal neuron activity *in vivo*.

## Discussion

In the present study, we applied MD simulations to predict the possible structural alterations produced by disease-associated point mutations that might be responsible for impairing the ability of gephyrin to form proper synaptic scaffolds. Of the six point mutations examined, A91T and G375D displayed reduced binding free energies for G-domain trimerization and E-domain dimerization, respectively, and failed to enhance the density of GABAergic synapses in cultured neurons, and hence were selected for extensive functional analyses. G375D, but not A91T, consistently exhibited loss-of-function phenotypes in all experimental settings. Although a previous report proposed impaired synaptic localization and decreased receptor binding as the mechanistic basis for the pathogenicity of the G375D mutant (Dejanovic et al., 2015), the present study provides a direct demonstration of the impact of this mutation on various gephyrin functions *in vivo*, including higher-order oligomer formation, interactions with key proteins of GABAergic synapses, and neuronal activity in the hippocampal DG area.

Our atomic-scale MD simulations indicated that the G375D mutation does not affect the structure of gephyrin in the E-domain dimer, but selectively reduces the binding free energy of the E-domain dimer compared with WT gephyrin (Figure 1). This implies that gephyrin G375D scaffolds are less stable than those formed by the WT protein, an interpretation consistent with the markedly reduced ability of G375D to form large intracellular aggregates upon expression in COS-7 cells (Figures 2C and 2D).

We found no synapse-promoting effects of G375D at GABAergic synapses in cultured hippocampal neurons (Figure 2). This result is consistent with the previously reported diffuse cytosolic distribution of gephyrin G375D in these cells (Dejanovic et al., 2015). We further observed that, in contrast to the case for gephyrin WT and A91T, overexpression of G375D in the hippocampal DG area of mice carrying floxed gephyrin alleles failed to rescue the region-specific loss of GABAergic synapses induced by Cre expression (Figures 3, S4 and S5). Similarly, the G375D mutant failed to normalize the increased susceptibilities to KA-induced seizures and abnormal epileptiform discharges observed in hippocampal DG-specific gephyrin-KO mice, whereas these pathophysiological phenotypes were rescued by gephyrin WT and A91T (Figure 4). Functional deficits at GABAergic synapses and reduced enzymatic activity of gephyrin have been proposed to account for G375D-mediated dysfunctions of gephyrin (Dejanovic et al., 2015). In the current study, we provide compelling evidence to support the conclusion that G375D influences the ‘structural’ integrity of gephyrin-containing protein complexes, as initially predicted by supercomputing-based simulations (Figure 1). However, we found no evidence for dominant-negative effects of G375D in cultured hippocampal neurons as reported by Dejanovic et al., 2015 (Figure 2), although it remains to be tested whether a lack of the dominant-negative effects by G375D mutation is also observed *in vivo*. Notably, using circular dichroism analyses, Dejanovic et al. found that bacterially produced recombinant gephyrin G375D proteins showed no folding defects (Dejanovic et al., 2015). In contrast, our results, obtained by transiently transfecting mammalian gephyrin G375D into HEK293T cells, unambiguously demonstrated impaired multimer formation of gephyrin, as revealed by semi-quantitative gel filtration analyses (Figures 1I and 1J). Because gephyrin is proposed to act as a scaffold that is critical for binding to and anchoring of other GABAergic synaptic proteins, including GABA_A_Rs, impaired formation of hexagonal lattices caused by the presence of G375D mutations might contribute to disorganization of GABAergic synapse structure, function and, possibly, plasticity (Alvarez, 2017; Choii and Ko, 2015; Groeneweg et al., 2018; Pizzarelli et al., 2019). To the best of our knowledge, this study is the first to test whether and how gephyrin missense mutations associated with neurological disorders contribute to dysregulation of structural and functional aspect of *in vivo* synapses. Although our various analyses did not reveal any structural or functional phenotypes for the other gephyrin missense mutations examined, the possibility remains that these mutations contribute to gephyrin-associated pathophysiology via as-yet unidentified mechanism(s).

Gephyrin interacts with various other synaptic proteins expressed exclusively at GABAergic synapses, such as GlyRs, the GABA_A_ receptor α3 subunit, Cbs, Nlgn2, GABARAP, Pin1, and IQSEC3, to coordinate inhibitory postsynaptic organization (Alvarez, 2017; Choii and Ko, 2015; Groeneweg et al., 2018; Pizzarelli et al., 2019). Armed with newly available antibodies specific for a subset of gephyrin-binding proteins (i.e. Cbs, Nlgn2 and IQSEC3), we examined whether gephyrin mutations influence the association of gephyrin with these proteins *in vivo*. Consistent with results from other assays, G375D, but not A91T, disengaged synaptic GABA_A_ receptors (monitored using GABA_A_Rγ2 antibodies) and Nlgn2, but not Cbs or IQSEC3, from gephyrin-containing complexes in hippocampal DG neurons (Figure 3). Although further in-depth analyses are required, our findings suggest that the G375D mutation likely induces structural changes in the E-domain of gephyrin, where the Gly375 residue is located, further influencing association with GABA_A_ receptors and Nlgn2. Because gephyrin binds Cbs and IQSEC3 through residues 319–329 and the G-domain, respectively (Kins et al., 2000; Um et al., 2016), it is plausible that G375D might exert little or no effect on interactions with Cbs and IQSEC3. On the other hand, because previous structural modeling and atomic force microscopy studies reported that gephyrin exists predominantly in a trimeric form (Sander et al., 2013), we are unable to completely rule out the possibility that G375D might affect gephyrin-binding affinity for Nlgn2 or GABA_A_ receptors. Whether G375D negatively affects GABAergic synaptic strength and postsynaptic assembly, in line with previous reports on the roles of gephyrin/Nlgn2 interaction (Antonelli et al., 2014; Poulopoulos et al., 2009), remains to be determined.

Diverse gephyrin mutations continue to be identified in human patients with variable neurological features and distinct neuropsychiatric disorders (Ko et al., 2015). Consistent with this, deletion of gephyrin selectively in forebrain mouse neurons markedly reduces GABA_A_R-positive puncta in the hippocampus and cortex, together with increased anxiety-like behavior and increased lethality presumably owing to prevalent epileptic seizures (O'Sullivan et al., 2016). Our results further showed that the loss of gephyrin in the hippocampal DG was sufficient to trigger epileptic seizures (Figure 4). The immediate goals of future studies should be to use conditional heterozygous gephyrin G375D knock-in mice to address whether G375D exhibits similar or distinct behavioral abnormalities compared with gephyrin forebrain cKO mice. In addition, considering the robustness of the effects of the G375D mutation, it would be extremely interesting to introduce induced neuronal cells, with the goal of replicating gephyrin G375D-associated disease phenotypes in human neurons.

### Limitations of the study

The current study clearly demonstrated that the gephyrin mutation, G375D, has impact on various gephyrin functions, including higher-order oligomer formation, interactions with Nlgn2 or GABA_A_ receptors, or network activity. However, it remains to be determined how other gephyrin mutations contribute to gephyrin-associated pathologies. In addition, it remains to be tested whether the lack of dominant-negative effects observed for G375D mutation is recapitulated *in vivo.* Lastly, future studies on higher-order oligomer formation of gephyrin *in vivo* are warranted to support the significance and mechanisms of G375D mutant-associated pathologies reported in the current study.

### Resource availability

#### Lead contact

Further information and requests for resources and reagents should be directed to and will be fulfilled by the Lead Contact, Ji Won Um (jiwonum@dgist.ac.kr).

#### Materials availability

All unique/stable reagents generated in this study are available from the Lead contact upon reasonable request and with a completed Materials Transfer Agreement.

#### Data and code availability

This study did not generate data sets.

## Methods

All methods can be found in the accompanying Transparent Methods supplemental file.

## Supplemental information

Document S1.Transparent methods and Figures S1–S6

## Footnotes

Supplemental Information can be found online at https://doi.org/10.1016/j.isci.2021.102037.
