# Supplementary material for: Impaired formation of high-order gephyrin oligomers underlies gephyrin dysfunction-associated pathologies
Source: iScience. 2021 Jan 7;24(2):102037. doi: 10.1016/j.isci.2021.102037 (PMC7822942; doi:10.1016/j.isci.2021.102037)
Supplement: Document S1.Transparent methods and Figures S1–S6 [file mmc1.pdf]

iScience, Volume *24*

Supplemental Information

Impaired formation of high-order gephyrin oligomers underlies gephyrin dysfunction-associated pathologies

Seungjoon Kim, Mooseok Kang, Dongseok Park, Ae-Ree Lee, Heinrich Betz, Jaewon Ko, Iksoo Chang, and Ji Won Um

SUPPLEMENTAL FIGURES

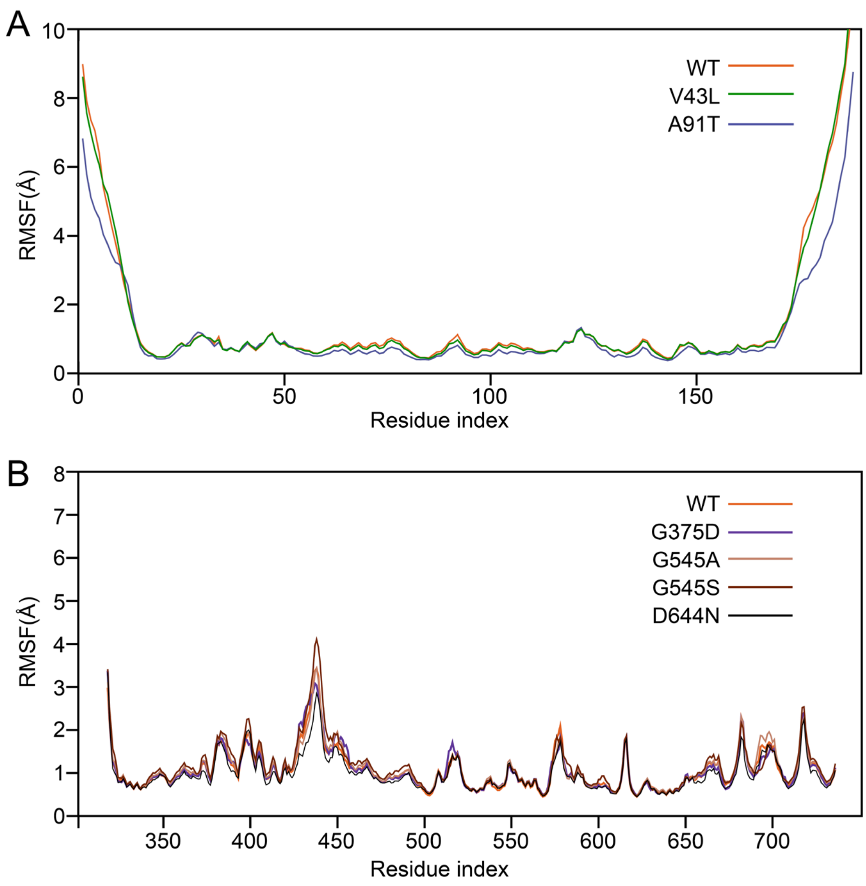

Supplemental Figure S1. Root-Mean-Square-Fluctuation (RMSF) of the Coordinate for Residues in Structures of Gephyrin WT and Mutants by MD Simulation (Related to *Figure 1*).

(**A**) Backbone RMSF of residues in the G-domain during a 0.5–1 μs time window from G-domain trimer MD simulation.

(**B**) Backbone RMSF of residues in the E-domain during a 0.5–1 μs time window from E-domain dimer MD simulation.

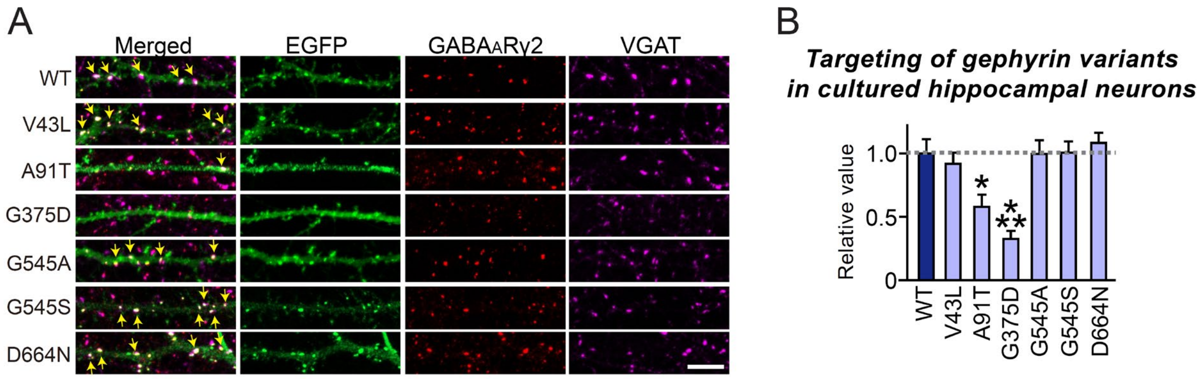

Supplemental Figure S2. GABAergic Synaptic Targeting of Gephyrin WT or Mutants in Cultured Hippocampal Neurons (Related to *Figure 2*).

(**A**) Representative images of cultured hippocampal neurons transfected at DIV10 with EGFP-tagged gephyrin WT or mutants, and analyzed at DIV14 by triple-immunofluorescence staining using anti-GABA$_A$Rγ2 (red), anti-VGAT (magenta) and anti-EGFP (green) antibodies. Yellow arrows mark colocalization of gephyrin with both GABA$_A$Rγ2 and VGAT puncta. Scale bar, 10 µm (applies to all images).

(**B**) Summary graphs showing inhibitory synaptic targeting of gephyrin variants in cultured hippocampal neurons. Data are presented as means ± SEMs from three independent experiments (2–3 dendrites per transfected neurons were analyzed and group-averaged; **p* < 0.05, ****p* < 0.001; non-parametric ANOVA with Kruskal-Wallis test followed by *post hoc* Dunn's multiple comparison test).

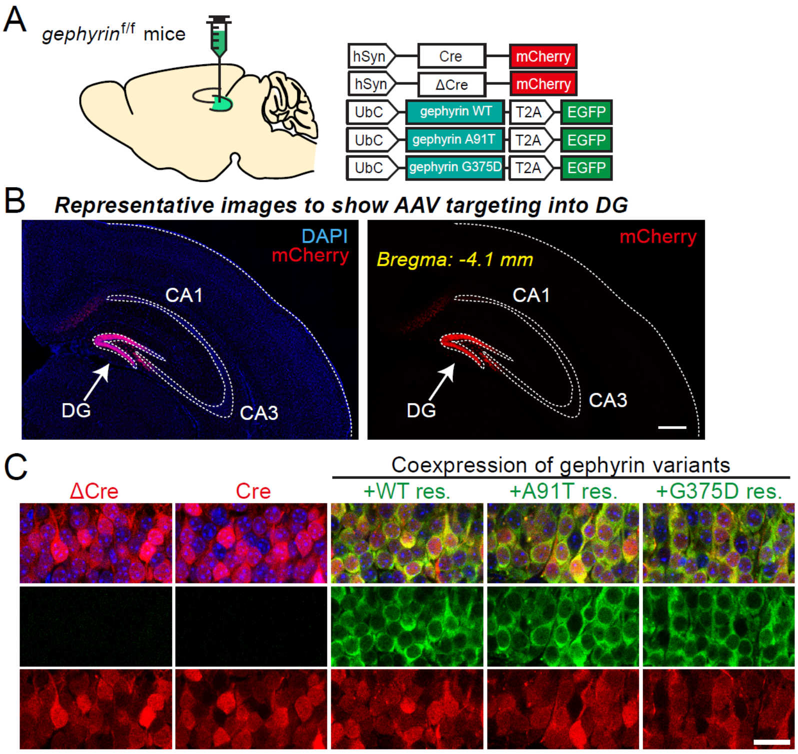

Supplemental Figure S3. Targeting of AAVs Expressing Gephyrin Variants into the Hippocampal DG of Gephyrin$^{f/f}$ Mice (Related to *Figures 3* and *4*).

(**A**) Schematic diagram of AAV vectors expressing Cre or ΔCre and WT gephyrin and its mutants (A91T and G375D) used for stereotactic injection into the DG of gephyrin floxed mice.

(**B**) Representative brain sections illustrating the precise targeting of adeno-associated virus (AAV) for expression of Cre recombinase in the DG. Scale bar: 500 µm.

(**C**) Representative images of hippocampal DG regions 2 weeks after stereotactic infection of gephyrin floxed mice with the indicated AAVs, followed by monitoring of infected neurons to validate the expression of rescue AAVs. Note that virus coinfection efficiency was 98.05 ± 0.70% for Cre and gephyrin WT, 97.58 ± 0.65% for Cre and gephyrin A91T, and 97.95 ± 0.79% for Cre and gephyrin G375D. Scale bar: 20 µm.

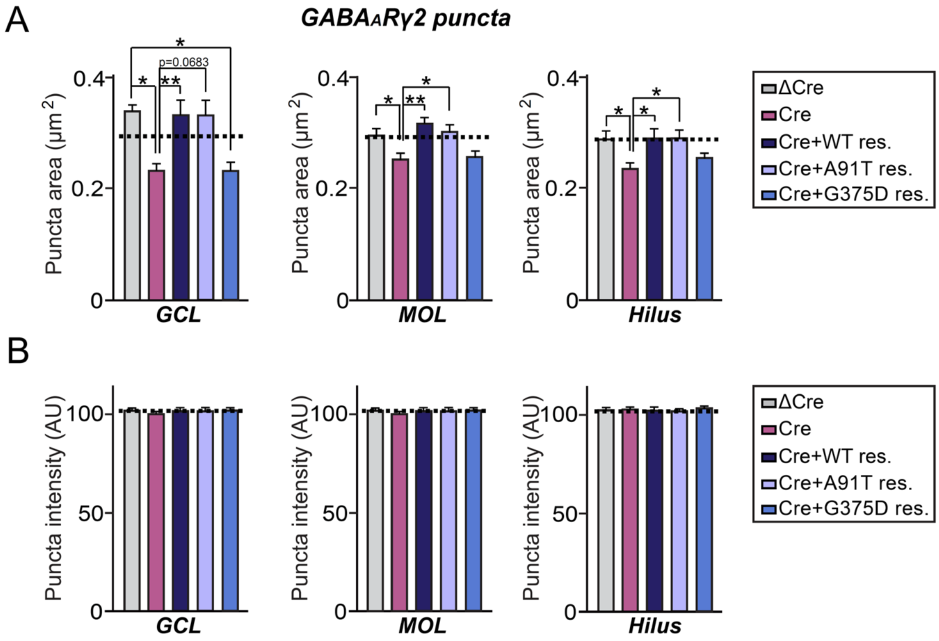

Supplemental Figure S4. Quantification of $GABA_AR\gamma2$-positive Puncta Area and Intensity in Gephyrin-deficient DG Neurons (Related to *Figure 3*).

(**A** and **B**) Quantification of the size (**A**), and intensity (**B**) of $GABA_AR\gamma2^+$ puncta. Data are presented as means ± SEMs (n = 4 mice/group after averaging data from 5 sections/mouse; *$p < 0.05$, **$p < 0.01$; non-parametric ANOVA with Kruskal-Wallis test followed by *post hoc* Dunn's multiple comparison test).

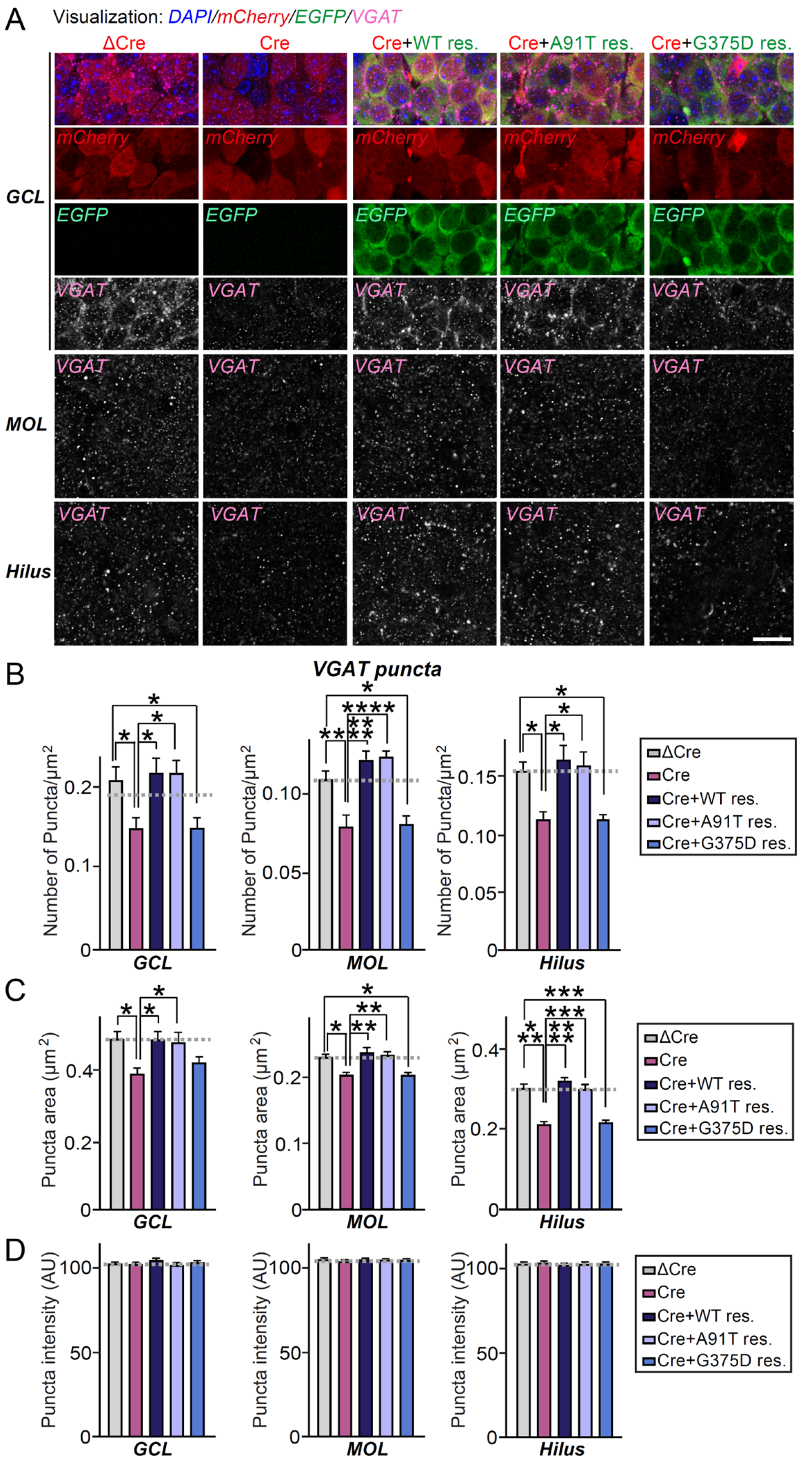

Supplemental Figure S5. Quantification of VGAT-positive Puncta in Gephyrin-deficient DG Neurons (Related to *Figure 3*).

(**A**) Representative images showing VGAT^{+} puncta in the DG of mice stereotactically injected with the indicated AAVs. Scale bar, 20 µm (applies to all images). Abbreviations: MOL, molecular layer; GCL, granule cell layer.

(**B-D**) Quantification of the density (**B**), size (**C**), and intensity (**D**) of VGAT+ puncta. Data are presented as means ± SEMs (n = 4 mice/group after averaging data from 5 sections/mouse; *$p < 0.05$, **$p < 0.01$, ***$p < 0.001$, ****$p < 0.0001$; non-parametric ANOVA with Kruskal-Wallis test followed by *post hoc* Dunn's multiple comparison test).

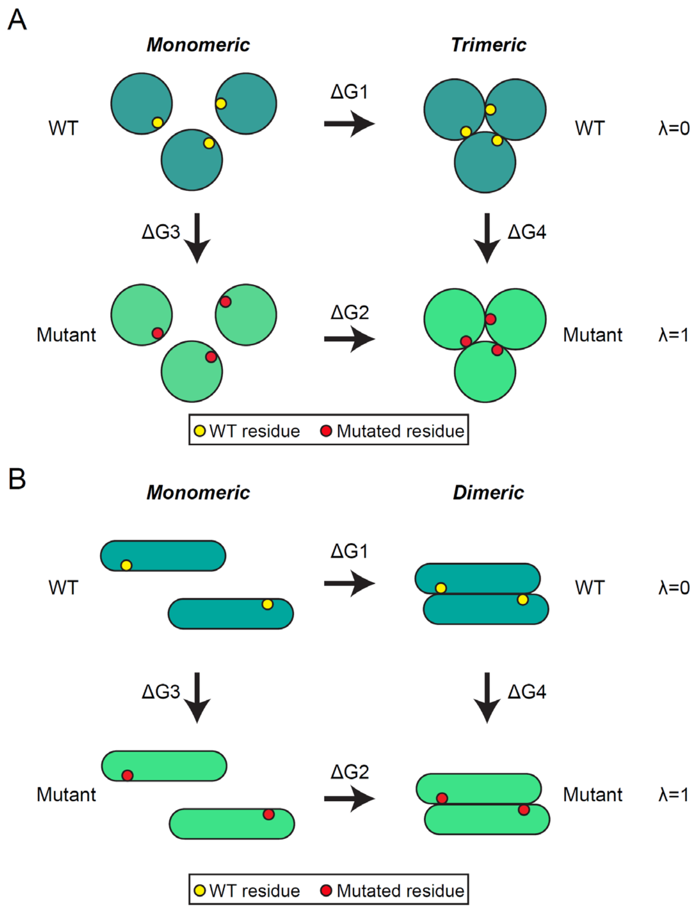

Supplemental Figure S6. Thermodynamic Cycle for Estimating Differences in Binding Free Energy, Calculated Using TI (Related to *Figure 1*).

(**A**) G-domain trimer.

(**B**) E-domain dimer.

TRANSPARENT METHODS

KEY RESOURES TABLE

REAGENT or RESOURCE	SOURCE	IDENTIFIER
**Antibodies**		
Rabbit polyclonal Anti-IQSEC3	Jaewon Ko's lab	JK079; RRID: AB_2687864
Mouse monoclonal Anti-Gephyrin	Synaptic Systems	Cat #147 111; RRID: AB_2619837
Rabbit monoclonal Anti-GABA$_A$Rγ2	Synaptic Systems	Cat #224 003; RRID: AB_2263066
Rabbit polyclonal Anti-VGAT	Synaptic Systems	Cat #131 003; RRID: AB_887869
Mouse monoclonal Anti-VGAT	Synaptic Systems	Cat #131 011; RRID: AB_887868
Goat polyclonal Anti-EGFP	Rockland	Cat #ab5450; RRID: AB_218182
Rabbit polyclonal Anti-Nlgn2	Synaptic Systems	Cat#129 202; RRID: AB_993011
Rabbit polyclonal Anti-Collybistin	Synaptic Systems	Cat#261 003; RRID: AB_2619977
Rabbit monoclonal Anti-TrkC	Cell Signaling	C44H5; RRID: AB_2155283
Rabbit polyclonal Anti-PSD-95	Jaewon Ko's lab	JK016; RRID: AB_2722693
FITC-conjugated Donkey Anti-goat IgG antibody	Jackson ImmunoResearch Laboratories	Cat# 705-007-003; RRID: AB_2340403
Cy3-conjugated Donkey Anti-Rabbit IgG antibody	Jackson ImmunoResearch Laboratories	Cat #711-165-152; RRID: AB_2307443
Cy3-conjugated Donkey Anti-Mouse IgG antibody	Jackson ImmunoResearch Laboratories	Cat #715-165-150; RRID: AB_2340813
Alexa Fluor® 647-conjugated Goat Anti-mouse IgG antibody	ThermoFisher Scientific	Cat #A28181; RRID: AB_2536165
Alexa Fluor® 647-conjugated Goat Anti-rabbut IgG antibody	ThermoFisher Scientific	Cat #A27040; RRID: AB_2536101
**Chemicals, Peptides, and Recombinant Proteins**		
Kainic acid	Sigma	Cat #K0250
Neurobasal medium	ThermoFisher Scientific	Cat #21103049
B-27 supplement (50X)	ThermoFisher Scientific	Cat #17504-044
Penicillin/Streptomycin	ThermoFisher Scientific	Cat #15140122
HBSS (Hanks' Balanced Salt Solution)	ThermoFisher Scientific	Cat #14065056
GlutaMax Supplement	ThermoFisher Scientific	Cat #35050061
FBS (Fetal Bovine Serum)	WELGENE	Cat #PK004-01

Sodium pyruvate	ThermoFisher Scientific	Cat #11360070
Poly-D-lysine hydrobromide	Sigma	Cat #P0899
Vectashield mounting medium	Vector Laboratories	Cat #H-1200
2,2,2-Tribromoethyl alcohol	Sigma	Cat #T48402
Tert-Amyl alcohol	Sigma	Cat #240846
**Experimental Models: Cell Lines**		
Cultured neuronal cells (from rat embryos)	N/A	N/A
HEK 293T cells	ATCC	Cat # CRL-3216
**Experimental Models: Organisms/Strains**		
Mouse: Gephyrin floxed mice		O'Sullivan et al., 2016
**Recombinant DNA**		
L313-Ub-gephyrin WT	This study	N/A
L313-Ub-gephyrin A91T	This study	N/A
L313-Ub-gephyirn G375D	This study	N/A
pEGFP-C2-gephyrin WT	This study	N/A
pEGFP-C2-gephyrin A91T	This study	N/A
pEGFP-C2-gephyrin G375D	This study	N/A
pEGFP-C2-gephyrin G545A	This study	N/A
pEGFP-C2-gephyrin G545S	This study	N/A
pEGFP-C2-gephyrin D664N	This study	N/A
pAAV2/9-gephyrin WT-T2A-EGFP	This study	N/A
pAAV2/9-gephyrin A91T-T2A-EGFP	This study	N/A
pAAV2/9-gephyrin G375D-T2A-EGFP	This study	N/A
pAAV-hSyn-ΔCre-mCherry	A gift from Dr. Thomas Südhof (Stanford University)	N/A
pAAV-hSyn-Cre-mCherry	A gift from Dr. Thomas Südhof (Stanford University)	N/A
**Software and Algorithms**		
MetaMorph	Molecular Devices	https://www.moleculardevices.com
GraphPad Prism 7.0	GraphPad	https://www.graphpad.com
AMBER18	D.A. Case et al., 2018	http://ambermd.org/
VMD 1.9.3	Humphrey et al., 1996	https://www.ks.uiuc.edu/Research/vmd/

EXPERIMENTAL MODEL AND SUBJECT DETAILS

Cell Culture

HEK293T cells and COS7 cells were cultured in Dulbecco's Modified Eagle's Medium (DMEM; WELGENE) supplemented with 10% fetal bovine serum (FBS; Tissue Culture Biologicals) and 1% penicillin-streptomycin (Thermo Fisher) at 37 °C in a humidified 5% CO2 atmosphere. All procedures were performed according to the guidelines and protocols for rodent experimentation approved by the Institutional Animal Care and Use Committee of DGIST.

Animals

Conditional gephyrin knockout mice (gephyrinflox/flox) were previously described (O'Sullivan et al., 2016). Mice were housed and bred at the animal facility of Daegu Gyeongbuk Institute of Science and Technology (DGIST), and their experimental uses were approved by the Institutional Animal Care and Use Committee of DGIST. All C57BL/6N mice were maintained and handled in accordance with protocols (DGIST-IACUC-19052109-00) approved by the Institutional Animal Care and Use Committee of DGIST under standard, temperature-controlled laboratory conditions. All experimental procedures were performed on male mice. Pregnant rats (Daehan Biolink) were used to prepare in vitro cultures of dissociated hippocampal neurons. All procedures were conducted according to the guidelines and protocols for rodent experimentation approved by the Institutional Animal Care and Use Committee of DGIST. Mice were kept on a 12:12 light/dark cycle (lights on at 7:00 am), and received water and food ad libitum.

METHOD DETAILS

Construction of Gephyrin 3D Structures. Previously reported structures of the rat gephyrin G-domain monomer (PDB ID, 1IHC; UNP ID, Q03555; residues 2–188) (Sola et al., 2001), human gephyrin G-domain trimer (PDB ID, 1JLJ; UNP ID, Q9NQX3; residues 1–181) (Schwarz et al., 2001), and rat gephyrin E-domain dimer (PDB ID, 4TK1; UNP ID, Q03555; residues 318–736) (Maric et al., 2014) were used in MODELLER (Webb and Sali, 2016) to fill in 3D structures for missing residues and reconstruct the full structures of G-domain trimers and E-domain dimers. The locations of missing residues were at the N- and C-termini of both domains and residues 431-440 of the E-domain. After building the structure of missing residues, loops were refined with an automatic loop refinement module with using the 'refine.fast' option in MODELLER. The C-domain of gephyrin is intrinsically structurally disordered, and thus is not compatible with modeling and simulation. Thus, simulations were performed only for structured regions of gephyrin (i.e., G-domain trimer and E-domain dimer). Mutant structures were generated by MD simulation using WT structures as templates. Figures for protein structures were created using VMD software (Humphrey et al., 1996).

Molecular Dynamic (MD) Simulations and Thermodynamic Integration (TI). MD simulations were preformed using PMEMD.CUDA (Salomon-Ferrer et al., 2013) in the AMBER18 package (D.A. Case, 2018) with the ff14SB force field (Maier et al., 2015). Gephyrin G-domain trimers and E-domain dimers were explicitly solvated with TIP3P water molecules (Jorgensen et al., 1983) in a rectangular box; a distance of 12 Å from the protein to the edge of the solvent box was chosen, and periodic boundary conditions were applied. Sodium ions were added to neutralize the system. The WT consisted of 85,173 atoms in the G-domain trimer and 104,523 atoms in the E-domain dimer. The particle-mesh Ewald (PME) method was applied for treatment of long-range electrostatic interactions, and a 9.0 Å force-shifted cutoff was used for short-range non-bonded interactions. The equilibrium bond length of hydrogen atoms was constrained using the SHAKE algorithm (Ryckaert et al., 1977); 2,500 steps of steepest decent minimization followed by 2,500 steps for conjugate gradient minimization were

performed. The systems were subsequently subjected to a 25-ps heating process in which the temperature was raised gradually from 10 K to 298 K with an NVT ensemble. After heating, a 25-ps equilibrium process using an NPT ensemble was applied. Production runs were carried out for 1 μs, with a 2 fs time step and applying the NPT ensemble. Temperature and pressure were controlled with a Langevin dynamic thermostat using a collision frequency of 1 ps−1 and a weak-coupling barostat with a coupling constant of 0.5 ps. All trajectories were recorded every 10 ps intervals. Each system was simulated for 5 independent trajectories, and a total of 40 trajectories were produced. The trajectories were analyzed using CPPTRAJ tools in the AmberTools package (D.A. Case, 2018). The backbone RMSF (root-mean-square-fluctuation) was calculated for backbone atoms (N, Cα, C) of gephyrin residues in 0.5~1 μs time windows of trajectories using the 'atomicfluc' command with the 'byres' option in CPPTRAJ (**Fig. S1**). TI-MD simulations were performed using the AMBER18 package with ff14SB force field (Lee et al., 2017; Mermelstein et al., 2018), and binding free energy differences were calculated using a thermodynamic cycle in conjunction with the condition of equal energy differences (**Fig. S6**). ΔG1 (ΔG2) is defined as the binding free energy between monomeric and multimeric states of the WT or mutant; ΔG3 (ΔG4) is the free energy difference between WT and mutant monomers (multimer). The thermodynamic equality is ΔG1+ΔG4-ΔG2-ΔG3 = 0. To obtain the binding free energy differences between WT and mutants, we can arrange the equation for the thermodynamic cycle as ΔG2-ΔG1 = ΔG4-ΔG3 = ΔΔG. The TI method, which uses a mixed potential function, was applied for calculating ΔG3 and ΔG4, and softcore potentials were used for smoothing the appearance and disappearance of atoms in van der Waals and electrostatic interactions in the hybrid state. In the TI calculation, the following Hamiltonian was used:

$$H_{\lambda} = (1-\lambda)H_{\mathrm{WT}} + \lambda H_{\mathrm{mutant}}$$

where λ is the weight of the mutant state and varies from 0 to 1. For example, if λ is 0.4, the mixed state has 60% WT structure and 40% mutant structure at the same position. As a result, the final structure of the hybrid state has mixed potential. The equations for vdW and electrostatic potential in softcore as follows:

$$V_{\text{WT,disappearing}}^{\text{vdW}} = 4\varepsilon(1-\lambda)\left[\frac{1}{\left[a\lambda + \left(\frac{r_{ij}}{\sigma}\right)^6\right]^2} - \frac{1}{a\lambda + \left(\frac{r_{ij}}{\sigma}\right)^6}\right]$$

$$V_{\text{mutant,appearing}}^{\text{vdW}} = 4\varepsilon\lambda\left[\frac{1}{\left[a(1-\lambda) + \left(\frac{r_{ij}}{\sigma}\right)^6\right]^2} - \frac{1}{a(1-\lambda) + \left(\frac{r_{ij}}{\sigma}\right)^6}\right]$$

$$V_{\text{WT,disappearing}}^{\text{elec}} = (1-\lambda)\frac{q_i q_j}{4\pi\varepsilon\sqrt{\beta\lambda + r_{ij}^2}}$$

$$V_{\text{mutant,appearing}}^{\text{elec}} = \lambda\frac{q_i q_j}{4\pi\varepsilon\sqrt{\beta(1-\lambda) + r_{ij}^2}}$$

We constructed the coordinate and parameter files for the hybrid state using the 'tleap' program. We also modified parameter files for mutation points at each chain using the 'parmed' module in AMBER. The major input file options for AMBER TI calculations are icfe = 1 (free energy calculation), ifsc =1 (softcore potential), scalpha = 0.5 (softcore potential parameter α), scbeta = 12.0 (softcore potential parameter β), temp0 = 300 (temperature, K) and clambda (λ). The free energy difference (ΔG) was calculated using Gaussian quadrature according to following formula:

$$\Delta G = \int_0^1 \langle\frac{\partial v}{\partial \lambda}\rangle_\lambda \approx \sum_i w_i \langle\frac{\partial v}{\partial \lambda}\rangle_i$$

We used a 9-point quadrature, with λ set to 0.01592, 0.08198, 0.19331, 0.33787, 0.5, 0.66213, 0.80669, 0.91802 and 0.98408, and weights of 0.04064, 0.09032, 0.13031, 0.15617, 0.16512, 0.15617, 0.13031, 0.09032 and 0.04064, respectively. A 5-ns TI simulation was performed for each λ value and 5 ensembles for each system. The binding free energy differences (ΔΔG) were applied cumulative average over course of the simulation and averaged for 5 trajectories of each gephyrin mutant.

Construction of Expression Vectors. ***1. Gephyrin***. pEGFP-Gephyrin wild-type (WT) was previously described (Papadopoulos et al., 2017). EGFP-Gephyrin A91T and pEGFP-Gephyrin G375D mutants were generated with a site-directed mutagenesis kit (Stratagene) using pEGFP-Gephyrin WT as a template. AAVs encoding full-length gephyrin WT, A91T or G375D were generated by PCR amplification of the corresponding pEGFP-gephyrin constructs and subsequent subcloning into *Xba*I and *BamH*I sites of the pAAV-T2A-EGFP vector (Kim et al., 2020). Gephyrin WT was generated by PCR amplification of the full-length region and subsequent subcloning into the L-313 vector at *EcoR*I and *BamH*I sites. pL313-Gephyrin A91T and G375D were generated with a site-directed mutagenesis kit (Stratagene) using pL313-Gephyrin WT as a template. ***2. Others***. The plasmids pAAV-hSyn-ΔCre-mCherry and pAAV-hSyn-Cre-mCherry were kindly provided by Dr. Thomas C. Südhof (Stanford University, Palo Alto, CA, USA).

Antibodies. The following commercially available antibodies were used: goat polyclonal anti-EGFP (Rockland), rabbit polyclonal anti-GABA$_A$Rγ2 (Synaptic Systems), rabbit polyclonal anti-Nlgn2 (Synaptic Systems), rabbit polyclonal anti-Collybistin (Synaptic Systems), mouse monoclonal anti-Gephyrin (clone 3B11; Synaptic Systems), rabbit polyclonal anti-VGAT (Synaptic Systems), mouse monoclonal anti-VGAT (Synaptic Systems), rabbit monoclonal anti-TrkC (C44H4; Cell Signaling), rabbit polyclonal anti-PSD-95 (JK016; Um et al., 2016) and rabbit polyclonal anti-IQSEC3 (JK079; (Kim et al., 2020)).

Gel Filtration Chromatography. HEK293T cells transfected with the indicated gephyrin expression vectors were harvested in phosphate-buffered saline (PBS), washed once with PBS, homogenized with an ultrasonicator in 1 ml of buffer A (50 mM Tris-HCl, pH 7.5; 150 mM NaCl; 5 mM DTT), and centrifuged at 10,000 × g for 15 minutes. The resulting supernatant was applied to a Superdex 200 10/300 GL column attached to an AKTA Purifier FPLC (GE Healthcare) and eluted with PBS containing 0.2 μm filtered 0.05% bovine serum albumen (BSA) at a flow rate of 0.5 ml/min. Aliquots (100 μl) of each fraction were collected and immunoblotted using anti-gephyrin antibodies. Globular protein size

standards (Sigma), including thyroglobulin (670 kDa), apoferritin (480 kDa), β-Amylase (223.8 kDa), alcohol dehydrogenase (141 kDa), bovine serum albumin (66 kDa), and ovalbumin (43 kDa) were run in parallel under identical experimental settings.

Neuron Culture, Transfections, Imaging, and Quantitation. Cultured hippocampal neurons were prepared from E18 rat brainscultured on coverslips coated with poly-L-lysine, and grown in Neurobasal medium supplemented with B-27 (Invitrogen), 0.5% FBS, 0.5 mM GlutaMax (Invitrogen), and sodium pyruvate (Invitrogen). For overexpression of gephyrin in cultured neurons, hippocampal neurons were transfected with pEGFP-Gephyrin WT or the indicated point mutants, or with EGFP (Control) using CalPhos Kit (Clontech) at DIV10 and immunostained at DIV14. For immunocytochemistry, cultured neurons were fixed with 4% paraformaldehyde/4% sucrose, permeabilized with 0.2% Triton X-100 in PBS, immunostained with primary antibodies as indicated, and detected with Cy3- and fluorescein isothiocyanate (FITC)-conjugated secondary antibodies (Jackson ImmunoResearch). Images were acquired using a confocal microscope (LSM800, Carl Zeiss) with a 63 x objective lenses; all image setting were kept constant. Z-stack images were converted to maximal projection and analyzed to obtain the size, intensity, and density of puncta immunoreactivities of marker proteins. Quantification was performed in a blinded manner using MetaMorph software (Molecular Devices). For quantification of GABAergic synaptic targeting of EGFP-gephyrin, overlaying red ($GABA_AR\gamma2$) and magenta (VGAT) color images were obtained using the color threshold function in ImageJ. Regions of interests (ROIs) from distal dendrites were defined according to pEGFP-signals. The degree of colocalization of green (pEGFP-gephyrin) and overlaid red ($GABA_AR\gamma2$) and magenta (VGAT) in the ROIs was calculated using the colocalization function in MetaMorph. The value for WT gephyrin was normalized to 1.

Production of Recombinant Adeno-associated Viruses (AAVs). Recombinant AAVs were packaged with high efficiency using pHelper and AAV1.0 (serotype 2/9) capsids. HEK293T cells were cotransfected with pHelper and pAAV1.0, together with pAAV-hSyn-ΔCre-EGFP, pAAV-hSyn-Cre-EGFP, pAAV-hSyn-ΔCre-mCherry, pAAV-hSyn-Cre-mCherry, pAAV-Gephyrin WT-T2A-GFP, pAAV-

Gephyrin A91T-T2A-EGFP, or pAAV-Gephyrin G375D-T2A-EGFP. Transfected HEK293T cells were harvested 72–108 hours post transfection. After addition of 0.5 M EDTA to the medium, cells were washed three times with PBS and collected by centrifugation. Cells were then resuspended in PBS and lysed by subjecting to four freeze-thaw cycles in an ethanol/dry ice bath (7 minutes each) and 37°C water bath (5 minutes). Lysates were centrifuged, and supernatants were collected and incubated with a solution containing 40% poly(ethylene glycol) (Sigma) and 2.5 M NaCl on ice for 1 hour and centrifuged at 2000 rcf for 30 minutes. The pellets were resuspended in HEPES buffer (20 mM HEPES pH 8.0, 115 mM NaCl, 1.2 mM $CaCl_2$, 1.2 mM $MgCl_2$, 2.4 mM KH_2PO_4), mixed with chloroform, and centrifuged at 400 rcf for 10 minutes. The supernatant was collected and concentrated using Amicon Ultra Centrifugal Filters (0.5 ml, 3K MWCO; Millipore). Viruses were assessed for infectious titer by RT-PCR, and used for infections at 10^{10}–10^{12} infectious units/μl.

Stereotactic Surgery. For stereotaxic delivery of recombinant AAVs, 6–week-old gephyrin floxed mice were anesthetized by inhalation of isoflurane (3–4%) or intraperitoneal injection of 2% 2,2,2-tribromoethanol (Sigma) in saline, and secured in a stereotaxic apparatus. Viral solutions were injected with a Hamilton syringe using a Nanoliter 2010 Injector (World Precision Instruments) at a flow rate of 50 nl/min (injected volume, 0.3 μl). The coordinates used for stereotaxic injections into the hippocampal DG of mice were as follows: anteroposterior (AP), -2.1 mm; medial–lateral (ML), ± 1.2 mm; and dorsal–ventral (DV), 2.2 mm from bregma. Each injected mouse was returned to its home cage and used for scoring seizure-like behaviors, immunohistochemical analyses, or electrophysiological recordings after 2 weeks.

***In Vivo* Coimmunoprecipitation Assays.** Brains from 6–week-old gephyrin floxed mice expressing pAAV-Cre or pAAV-Cre together with pAAV-Gephyrin WT, A91T or G375D (delivered by stereotactic injection) were homogenized in 10 ml of ice-cold homogenization buffer consisting of 320 mM sucrose, 5 mM HEPES-NaOH (pH 7.5), 1 mM EDTA, 0.2 mM PMSF, 1 μg/ml aprotinin, 1 μg/ml leupeptin, 1 μg/ml pepstatin, and 1 mM Na_3VO_4. The homogenized tissue was centrifuged at 1000 × g for 10

minutes, after which the supernatant was centrifuged at 15,000 × g for 20 minutes. The pellets were homogenized in buffer consisting of 20 mM HEPES-NaOH (pH 7.5), 0.15 M NaCl, 2 mM $CaCl_2$, 2 mM $MgCl_2$, 0.2 mM PMSF, 1 µg/ml aprotinin, 1 µg/ml leupeptin, 1 µg/ml pepstatin, and 1 mM Na_3VO_4. Triton X-100 was added to a final concentration of 1% (w/v) and dissolved with constant stirring at 4°C for 1 hour. Supernatants obtained after centrifugation at 15,000 × g for 20 minutes were incubated with anti-gephyrin or anti-$GABA_AR\gamma2$ antibody overnight at 4°C, followed by addition of 30 µl of a 1:1 suspension of protein G-Sepharose (Incospharm Corporation), after which the mixture was incubated for 2 hours at 4°C with gentle rotation. The beads were pelleted and washed three times with lysis buffer (20 mM HEPES-NaOH pH 7.5, 0.15 M NaCl, 2 mM $CaCl_2$, 2 mM $MgCl_2$, 0.2 mM PMSF, 1 µg/ml aprotinin, 1 µg/ml leupeptin, 1 µg/ml pepstatin, and 1 mM Na_3VO_4). Immune complexes were then resolved by SDS-PAGE and immunoblotted with anti-gephyrin, anti-Nlgn2, anti-Collybistin, anti-IQSEC3, anti-PSD-95, anti-TrkC and anti-$GABA_AR\gamma2$ antibodies, each at a concentration of 1 µg/ml.

Immunohistochemistry. Six–week-old male C57BL/6N mice stereotactically injected with the indicated AAVs were anesthetized and immediately perfused, first with PBS for 3 minutes and then with 4% paraformaldehyde for 5 minutes. Brains were dissected out, fixed overnight in 4% paraformaldehyde, incubated overnight with 30% sucrose (in PBS), and then sliced into 30-µm-thick coronal sections using a vibratome (Model VT1200S; Leica Biosystems). Sections were permeabilized by incubating with 1% Triton X-100 in PBS containing 5% BSA and 5% horse serum for 30 minutes. For immunostaining, sections were incubated for 8–12 hours at 4 °C with primary antibodies in the same blocking solution. Sections were washed three times in PBS and incubated with the appropriate Cy3-, fluorescein isothiocyanate (FITC)-conjugated secondary antibodies (Jackson ImmunoResearch) or Alexa Fluor® 647-conjugated secondary antibodies (ThermoFisher Scientific) for 2 hours at room temperature. After three washes with PBS, sections were counterstained with DAPI (4’,6-diamidino-2-phenylinodole) and mounted onto glass slides (Superfrost Plus; Fisher Scientific) with VECTASHIELD mounting medium (H-1200; Vector Laboratories).

Seizure Behavior Scoring. Six-week-old male C57BL/6N mice stereotactically injected with the indicated AAVs were administered KA (15 mg/kg; Sigma Cat. No. K0250) or saline (control), and the resulting seizure behaviors were video-recorded for the next 2 hours. Seizure susceptibility was measured by rating seizures every 3 minutes on a scale of 0 to 5, as follows: 0, no abnormal behavior; 1, reduced motility and prostate position; 2, partial clonus; 3, generalized clonus including extremities; 4, tonic-clonic seizure with rigid paw extension; and 5, death.

Electroencephalography (EEG) Recordings and Analyses. For EEG recordings, 6–week-old male C57BL/6N mice stereotactically injected with the indicated AAVs were anesthetized with isoflurane (3-4%) and secured in a stereotaxic apparatus to prevent limb-withdrawal response to a noxious foot pinch. After injection of the indicated concentrated AAVs into the hippocampal DG of experimental mice prabove, four additional holes were drilled in the skull without puncturing the meninges, and electrodes were carefully attached with prefabricated headmounts and secured with dental acrylic. Recording electrodes were placed in the parietal lobe (AP -2.4 mm; ML ± 1.4 mm), and reference electrodes were placed in the occipital lobe. The LFP electrode was inserted at the DG (AP -2.4 mm; ML ± 1.3 mm, DV -2.2). After a 2-week recovery period, mice connected to the EEG/EMG three-channel monitoring system, composed of preamplifier and a commutator for data acquisition (Pinnacle Technology) with time-lock video recording, were transferred to an acrylic cage (25 × 25 × 45 cm). Each mouse was subjected to a 24-hour recording session for measuring spontaneous seizures and a 4-hour recording session for measuring KA-induced seizures. Data were recorded at a sampling rate of 2000 Hz, with application of a 100-Hz loss-pass filter. Off-line EEG analyses were performed manually in MatLab (Mathworks) using EEGlab (SCCN)) or LabChart 8.0 software (ADInstruments). For manual analyses of electrographic seizures, the following previously described criteria were used, with minor modifications (Baraban et al., 2009): grade I, basic background, no epileptiform spikes; grade II, mostly normal background, some high-voltage spikes; grade III, mostly abnormal background with low-frequency, high-voltage spiking; and grade IV, high-frequency, high-voltage, synchronized polyspike waves with amplitudes > 3-fold baseline. Only ictal seizures (grade III or IV) lasting longer than 3

seconds were included in the analysis. For automated quantification of interictal spikes, selected frequencies (0–60 Hz) were digitally filtered using low-pass filter in LabChart 8.0 software (ADInstruments), and amplitudes > 3-fold baseline discharges were automatically calculated using the native LabChart function.

QUANTIFICATION AND STATISTICAL ANALYSIS

Data Analysis and Statistics. All data are expressed as means ± SEM. All experiments were repeated using at least three independent cultures, and data were statistically evaluated using a Mann-Whitney *U* test, Kruskal-Wallis test (one-way ANOVA on ranks) followed by Dunn's pairwise *post hoc* test, as appropriate. Prism7.0 (GraphPad Software) was used for analysis of data and preparation of bar graphs. P-values < 0.05 were considered statistically significant (individual *p*-values are presented in figure legends).

SUPPLEMENTAL REFERENCES

D.A. Case, I.Y.B.-S., S.R. Brozell, D.S. Cerutti, T.E. Cheatham, III, V.W.D. Cruzeiro, T.A. Darden, R.E. Duke, D. Ghoreishi, M.K. Gilson, H. Gohlke, A.W. Goetz, D. Greene, R Harris, N. Homeyer, S. Izadi, A. Kovalenko, T. Kurtzman, T.S. Lee, S. LeGrand, P. Li, C. Lin, J. Liu, T. Luchko, R. Luo, D.J. Mermelstein, K.M. Merz, Y. Miao, G. Monard, C. Nguyen, H. Nguyen, I. Omelyan, A. Onufriev, F. Pan, R. Qi, D.R. Roe, A. Roitberg, C. Sagui, S. Schott-Verdugo, J. Shen, C.L. Simmerling, J. Smith, R. Salomon-Ferrer, J. Swails, R.C. Walker, J. Wang, H. Wei, R.M. Wolf, X. Wu, L. Xiao, D.M. York and P.A. Kollman (2018). AMBER 2018. University of California, San Francisco.

Humphrey, W., Dalke, A., and Schulten, K. (1996). VMD: visual molecular dynamics. J Mol Graph 14, 33-38, 27-38.

Jorgensen, W.L., Chandrasekhar, J., Madura, J.D., Impey, R.W., and Klein, M.L. (1983). Comparison of simple potential functions for simulating liquid water. J Chem Phys 79, 926-935.

Kim, S., Kim, H., Park, D., Kim, J., Hong, J., Kim, J.S., Jung, H., Kim, D., Cheong, E., Ko, J., et al. (2020). Loss of IQSEC3 Disrupts GABAergic Synapse Maintenance and Decreases Somatostatin Expression in the Hippocampus. Cell Reports 30.

Lee, T.S., Hu, Y., Sherborne, B., Guo, Z., York, D.M. (2017). Toward fast and accurate binding affinity prediction with pmemdGTI: an efficient implementation of GPU-accelerated thermodynamic integration. J Chem Theor Comput 13, 3077-3084

Maier, J.A., Martinez, C., Kasavajhala, K., Wickstrom, L., Hauser, K.E., and Simmerling, C. (2015). ff14SB: Improving the Accuracy of Protein Side Chain and Backbone Parameters from ff99SB. J Chem Theory Comput 11, 3696-3713.

Maric, H.M., Kasaragod, V.B., Hausrat, T.J., Kneussel, M., Tretter, V., Stromgaard, K., and Schindelin, H. (2014). Molecular basis of the alternative recruitment of GABA(A) versus glycine receptors through gephyrin. Nat Commun 5, 5767.

Mermelstein, D.J., Lin, C., Nelson, G., Kretsch, R., McCammon, J.A., Walker, R.C. (2018). Fast and flexible gpu accelerated binding free energy calculations within the amber molecular dynamics package. J Comput Chem 39, 1354-1358.

O'Sullivan, G.A., Jedlicka, P., Chen, H.X., Kalbouneh, H., Ippolito, A., Deller, T., Nawrotzki, R.A., Kuhse, J., Kalaidzidis, Y.L., Kirsch, J., et al. (2016). Forebrain-specific loss of synaptic GABAA receptors results in altered neuronal excitability and synaptic plasticity in mice. Mol Cell Neurosci 72, 101-113.

Papadopoulos, T., Rhee, H.J., Subramanian, D., Paraskevopoulou, F., Mueller, R., Schultz, C., Brose, N., Rhee, J.S., and Betz, H. (2017). Endosomal Phosphatidylinositol 3-Phosphate Promotes Gephyrin Clustering and GABAergic Neurotransmission at Inhibitory Postsynapses. J Biol Chem 292, 1160-1177.

Ryckaert, J.-P., Ciccotti, G., and Berendsen, H.J.C. (1977). Numerical integration of the cartesian equations of motion of a system with constraints: molecular dynamics of n-alkanes. Journal of Computational Physics 23, 327-341.

Salomon-Ferrer, R., Gotz, A.W., Poole, D., Le Grand, S., and Walker, R.C. (2013). Routine Microsecond Molecular Dynamics Simulations with AMBER on GPUs. 2. Explicit Solvent Particle Mesh Ewald. J Chem Theory Comput 9, 3878-3888.

Sola, M., Kneussel, M., Heck, I.S., Betz, H., and Weissenhorn, W. (2001). X-ray crystal structure of the trimeric N-terminal domain of gephyrin. J Biol Chem 276, 25294-25301.

Schwarz, G., Schrader, N., Mendel, R.R., Hecht, H.J., and Schindelin, H. (2001). Crystal structures of human gephyrin and plant Cnx1 G domains: comparative analysis and functional implications. J Mol Biol 312, 405-418.

Um, J.W., Choi, T.Y., Kang, H., Cho, Y.S., Choii, G., Uvarov, P., Park, D., Jeong, D., Jeon, S., Lee, D., et al. (2016a). LRRTM3 Regulates Excitatory Synapse Development through Alternative Splicing and Neurexin Binding. Cell Rep 14, 808-822.

Webb, B., and Sali, A. (2016). Comparative Protein Structure Modeling Using MODELLER. Curr Protoc Protein Sci 86, 2 9 1-2 9 37.
